# Genetic variability of human papillomavirus type 18 based on E6, E7 and L1 genes in central China

**DOI:** 10.1186/s12985-024-02424-9

**Published:** 2024-07-05

**Authors:** Ting Li, Zhiping Yang, Ping Luo, Yang Yang, Zicong Lin, Bing Mei

**Affiliations:** Department of Laboratory Medicine, Jingzhou Hospital Affiliated to Yangtze University, Jingzhou, 434020 China

**Keywords:** Human papillomavirus (HPV) 18, E6, E7 and L1 genes, Variants, Epitope prediction

## Abstract

**Background:**

High-risk human papillomavirus (HR-HPV) infection is an important factor for the development of cervical cancer. HPV18 is the second most common HR-HPV after HPV16.

**Methods:**

In this study, MEGA11 software was used to analyze the variation and phylogenetic tree of HPV18 E6-E7 and L1 genes. The selective pressure to E6, E7 and L1 genes was estimated using pamlX. In addition, the B cell epitopes of L1 amino acid sequences and T cell epitopes of E6-E7 amino acid sequences in HPV18 were predicted by ABCpred server and IEDB website, respectively.

**Results:**

A total of 9 single nucleotide variants were found in E6-E7 sequences, of which 2 were nonsynonymous variants and 7 were synonymous variants. Twenty single nucleotide variants were identified in L1 sequence, including 11 nonsynonymous variants and 9 synonymous variants. Phylogenetic analysis showed that E6-E7 and L1 sequences were all distributed in A lineage. In HPV18 E6, E7 and L1 sequences, no positively selected site was found. The nonconservative substitution R545C in L1 affected hypothetical B cell epitope. Two nonconservative substitutions, S82A in E6, and R53Q in E7, impacted multiple hypothetical T cell epitopes.

**Conclusion:**

The sequence variation data of HPV18 may lay a foundation for the virus diagnosis, further study of cervical cancer and vaccine design in central China.

## Background

Human papillomavirus (HPV) infects human epithelial cells, mainly in skin and mucous membrane. In women, HPV infections are associated with cervical cancer. At present, more than 200 HPV genotypes have been identified [1]. Over 99% of cervical cancers contain HPV DNA, and the proportion related to specific high-risk human papillomavirus (HR-HPV) types varies geographically and ethnically [2]. HR-HPV is classified into 15 types (HPV16, 18, 21, 33, 35, 39, 45, 51, 52, 56, 58, 59, 68, 73 and 82), among which HPV16 and HPV18 are two of the most prevalent types and account for about 70% of all HPV related cervical cancers [3]. Persistent HPV infection is the major cause of cervical cancer [4].

The genome of papillomavirus contains a double-stranded circular DNA of about 8 kb in size. The papillomavirus genome is generally divided into three distinct regions: early genes (E1, E2, E4, E5, E6, E7 and E8), late genes (L1 and L2) and the upstream regulatory region (URR) [3]. The overexpression of viral oncoproteins E6 and E7 is an important factor and prerequisite for the malignant transformation of cells [5]. The malicious growth of HPV-infected cancer cells is based on the continuous expression of the E6 and E7 oncogenes [6]. So, the E6 and E7 proteins are often considered the ideal targets for the development of therapeutic HPV vaccines [6–8]. The E6 and E7 oncoproteins play an important role in unregulated cell proliferation and cancer development, and the carcinogenic characteristics of HPV DNA depend largely on the function of E6 and E7 oncoproteins [9]. The E6 oncoprotein binds to the cellular ubiquitin ligase E6AP, and the E6/E6AP complex recruits and degrades P53 [10]. The E7 protein binds to the tumor suppressor pRB and degrades it, causing the release of E2F transcription factor which promotes the mitotic phase of cell cycle and induces excessive cell proliferation [9, 11]. The L1 and L2 genes encode viral capsid proteins, and purified L1 proteins can self-assemble into virus-like particles (VLPs) with high immunogenicity [12]. The process of L1 protein self-assembly in VLPs is influenced by the polymorphism of the viral L1 gene [13, 14]. VLPs are widely used in HPV prophylactic vaccines preparation, and the production of protective antibodies often requires intact VLPs involvement [15].

HPV18, which belongs to the alpha genus and the A7 species, can be divided into 3 lineages and 9 sublineages: (1) A, A1–A5; (2) B, B1–B3; (3) C [16]. In China, 16, 52, 58, 53 and 18 were the top five most common HPV genotypes [17]. It has been reported that in invasive cervical cancer, HPV16 and HPV18 were the most common types in all regions [18]. However, HPV variation and the development of cervical cancer also show significant genetic diversity and geographical characteristics [19]. At present, the research on HPV18 variation in central China is limited to some extent. The purpose of this study was to identify and analyze the sequence variation and corresponding amino acid variation of HPV18 E6-E7 and L1 genes in Jingzhou, central China, which may provide useful data for HPV prevention and treatment studies in specific populations in this region.

## Materials and methods

### Ethics statement

All patients provided informed consent, and formal informed consent was acquired. This study was approved by the Ethics Committee of Jingzhou Hospital Affiliated to Yangtze University, and all the work followed the ethics guidelines of the hospital. The ethical approval number is 2022–048-01. Data were analyzed anonymously.

### Specimen collection

In this study, samples of cervical exfoliated cell were collected from the female patients with possible HR-HPV infection through routine cervical screenings in Jingzhou Hospital Affiliated to Yangtze University from September 2019 to October 2023. 150 women (rang from 18 to 80 years old, mean, 45.44 ± 11.23) were infected with a single HPV18 infection. All the samples were temporarily stored at 4℃, and DNA extraction was done the same day.

### HPV DNA extraction and typing

Based on the method of magnetic beads, DNA was extracted using the nucleic acid extracting kit (Guangzhou Magen Biotechnology Co., Ltd.) according to the manufacturer’s instruction. The DNA extraction products were measured by real-time quantitative PCR according to the instruction of HR-HPV typing kit (Shanghai ZJ Bio-Tech Co., Ltd.) as described previously [20]. Both positive and negative controls were applied in whole PCR amplification process. Finally, DNA extraction products of single positive samples of HPV18 were selected and stored at − 80℃ for the follow-up experiments.

### PCR amplification and sequencing

HPV18 single positive samples were chosen and used to amplify the full length of E6, E7 and L1 genes. Amplification of HPV18 E6, E7and L1 genes were performed using type-specific primers, which were shown in Table 1. All primers were synthesized by Sangon Biotech (Shanghai). Each 25μL PCR reaction contained 15.875μL ddH_2_O, 2.5μL 10 × buffer (MgCl_2_) (Takara), 2.0μL dNTP (Takara), 1.25μL forward and reverse primers (20 μM) (Sangon Biotech), 0.125μL Taq polymerase (5U/L) (Takara), and 2.0μL DNA extraction. The PCR reaction conditions were as follows: initial denaturation at 95 °C for 3 min, followed by 35 cycles of 94 °C for 45 s, annealing at the primer-specific temperature for 45 s and 72 °C for 60 s, the final extension at 72 °C for 10 min. The primer-specific annealing temperatures for each gene were also showed in Table 1. PCR products were visualized by 2.5% agarose gel electrophoresis. PCR products with clear and single electrophoretic bands were then sent to Sangon Biotech for sequencing.

**Table 1:** The primers and annealing temperatures of HPV18 E6, E7 and L1 genes

Gene	Primer name	Primer position	Forward primer/ Reverse primer	Product size (bp)	Anneal (℃)
E6	E6-F	83	GTGAGAAACACACCACAATAC	531	57.4℃
	E6-R	613	CAATGTTGCCTTAGGTCCAT		
E7	E7-F	540	CGACAGGAACGACTCCAA	383	59.4℃
	E7-R	922	ATCAGCCATTGTTGCTTACT		
L1-1	L1-1-F	5266	CACGGAGGACAATGACTT	713	59.3℃
	L1-1-R	5978	AAATGGATGCCCACTAAGG		
L1-2	L1-2-F	5778	GGTGGCAATAAGCAGGATA	747	59.3℃
	L1-2-R	6524	GGAGTCAGAGGTAACAATAGA		
L1-3	L1-3-F	6420	ACTGTGCCTCAATCCTTAT	819	55.4℃
	L1-3-R	7238	CAACAACAACCATACATACC		

*Abbreviations*: *F* Forward primer, *R* Reverse primer

### Variation analysis

Sequences of amplified E6-E7 and L1 were aligned with prototype reference sequence (GenBank: AY262282) of HPV18 using MEGA11 software. The nucleotide variant positions of E6-E7 and L1 were numbered accordingly. NCBI BLAST was used to check for new variants. The nucleotide sequences were translated into protein sequences using MEGA11 for determination of amino acid changes caused by nucleotide changes. GOR4 was used to further predict the secondary structure of proteins. The online software SWISS-MODEL (https://swissmodel.expasy.org/) was used to analyze the effect of non-synonymous substitutions on the predicted conformations of epitopes.

### Phylogenetic analysis

Phylogenetic trees of HPV18 E6-E7 and L1 gene sequences were constructed respectively by the Maximum Likelihood method and Kimura 2-parameter model using MEGA11. Reference sequences that represent each HPV18 lineage were used to construct the distinct phylogenetic branches, which involving A1(AY262282), A2(EF202146), A3(EF202147), A4(EF202151), A5(GQ180787), B1(EF202155), B2(KC470225), B3(EF202152) and C(KC470229) [16].

### Selective pressure analysis

The CodeML program, based on Maximum likelihood method in pamlX software, was used to determine nonsynonymous/synonymous nucleotide divergence and positively selected sites of HPV18 E6, E7 and L1 sequences. The posterior probability of a positively selected site was calculated using the Bayes empirical Bayes method.

### Epitope prediction

The ABCpred server (https://webs.iiitd.edu.in/raghava/abcpred/ABC_help.html) was used to predict the B cell epitopes of HPV18 L1 reference and variant sequences according to the default parameters. The higher the predicted score, the stronger the affinity of the epitope.

The T cell epitopes of HPV18 E6 and E7 proteins were predicted based on MHC-I (MHC, major histocompatibility complex) and MHC-II alleles using the Immune Epitope Database Analysis (IEDB) Resource (https://www.iedb.org/). The IEDB site recommended selecting the default prediction method, setting the epitope length as “All Length” and the rest default parameters. HLA‐I (HLA, human leukocyte antigen) and HLA‐-III alleles of the average frequency > 5% in the Chinese Han population were selected. The selection of predicted epitopes was based on lower percentile values, with HLA-I epitopes < 1.0 and HLA-II epitopes < 5.0. A low percentile rank (PR) indicated strong binding to HLA molecules.

## Results

### HPV18 E6-E7 gene variation

Eighty-four HPV18 single positive samples were amplified and sequenced, among which 62 E6-E7 sequences and 58 L1 sequences were successfully obtained. The rest samples were excluded due to PCR or sequencing failure. Genetic variants of these sequences are shown in Tables 2 and 3, respectively.

**Table 2:** HPV18 E6-E7 nucleotide mutation sites

	**E6**						**E7**			**N**
**Positions**	**348**	**392**	**467**	**482**	**485**	**549**	**747**	**751**	**835**	**62**
Reference	T	T	T	A	T	C	G	C	C	52
18LX01	G	-	-	-	-	-	-	-	-	1
18LX02	-	-	-	-	-	-	A	-	-	1
18LX03	-	C	-	-	-	-	-	-	-	1
18LX04	-	-	-	-	C	A	-	T	-	1
18LX05	-	-	G	-	C	A	-	-	-	1
18LX06	-	-	-	-	-	-	-	-	T	1
18LX07	-	-	-	-	C	A	-	-	-	1
18LX08	-	-	-	-	C	A	-	T	-	2
18LX09	-	-	-	C	-	-	-	-	-	1
Reference AA	S	T	L	R	F	R	R	A	D	
AA Position	82	96	121	126	127	149	53	54	82	
AA Variant	A	-	-	-	-	-	Q	-	-	
Secondary structure	-	-	H	H	H	H	H	-	H	

The nucleotides matching the reference (GenBank: AY262282) are marked with a dash (-), *AA* Amino acid, *H* Helix

**Table 3:** HPV18 L1 nucleotide mutation sites

	**L1**																				**N**
**Positions**	**5503**	**5520**	**5580**	**5769**	**5774**	**5832**	**5875**	**5882**	**5920**	**5924**	**5942**	**6125**	**6401**	**6404**	**6430**	**6878**	**6921**	**6970**	**7045**	**7062**	**58**
Reference	G	A	A	C	A	A	C	T	C	A	T	A	A	A	A	A	G	A	A	C	1
18ME01	A	-	-	-	-	-	-	-	-	-	-	-	-	-	-	-	-	-	-	-	37
18ME02	A	-	-	-	-	C	-	-	-	C	-	-	-	-	-	-	-	-	-	-	4
18ME03	A	-	-	-	-	-	-	-	T	-	-	-	-	-	-	-	-	-	-	-	5
18ME04	A	-	-	-	-	-	-	-	-	-	-	-	-	G	-	-	-	-	C	-	1
18ME05	A	-	-	-	-	-	A	-	T	-	G	-	-	-	-	-	-	-	-	-	1
18ME06	A	-	-	-	C	-	-	-	-	-	-	-	-	-	-	-	-	-	-	-	1
18ME07	A	-	-	T	-	-	A	C	-	-	-	G	-	-	-	C	-	C	-	-	1
18ME08	A	-	-	-	-	-	A	-	T	-	-	-	G	-	C	-	-	-	-	-	3
18ME09	A	-	-	-	-	-	-	-	T	-	-	-	-	-	-	-	-	-	-	T	1
18ME10	A	-	G	-	-	-	-	-	-	-	-	-	-	-	-	-	-	-	-	-	1
18ME11	A	-	-	-	-	-	A	-	T	-	-	-	-	-	C	-	-	-	-	-	1
18ME12	A	G	-	-	-	-	-	-	-	-	-	-	-	-	-	-	A	-	-	-	1
Reference AA	R	S	R	P	A	R	T	I	A	G	G	K	R	A	Q	V	E	K	K	R	
AA Position	25	31	51	114	115	135	149	151	164	165	171	232	324	325	334	483	498	514	539	545	
AA Variant	Q	G	G	S	-	-	N	-	V	-	-	-	-	-	P	-	K	T	T	C	
Secondary structure	-	S	-	S	-	S	-	-	S	S	-	-	H	H	-	S	-	H	-	-	

The nucleotides matching the reference (GenBank: AY262282) are marked with a dash (-), *AA* Amino acid, *S* Strand, *H* Helix

Out of 62 E6-E7 sequences, 42 (67.74%) had cytopathological diagnosis, including 28 women diagnosed with cervicitis, 5 with CIN1, 2 with CIN2/3, 4 with cervical carcinoma and 3 with normal, respectively. Among the 62 E6-E7 sequences, 10 sequences had nucleotide variants, while the remaining 52 sequences were completely homologous to the reference sequence. The identical sequences represented a specific variant group, so E6‐E7 variant sequences were divided into 9 different variant groups denoted as 18LX01–18LX09 in this study, which had been submitted to GenBank (accession numbers OR880987–OR880995). In total, we found 9 nucleotide substitutions in E6-E7 sequences. Among them, 5 nucleotide substitutions were already reported: A482C, T485C, C549A, G747A and C751T [21–23], and 4 nucleotide substitutions were newly identified: T348G, T392C, T467G and C835T. 9 single nucleotide variants were found in E6-E7 sequences, including 2 non-synonymous variants T348G (S82A), G747A (R53Q) and 7 synonymous variants T392C, T467G, A482C, T485C, C549A, C751T, C835T. One non-synonymous variants (R53Q) were found in E7 sequences encoding the alpha helix. The detailed results were shown in Table 2.

### HPV18 L1 gene variation

Out of 58 L1 sequences, 39 (67.24%) had cytopathological diagnosis, including 29 women diagnosed with cervicitis, 3 with CIN1, 3 with CIN2/3, 2 with cervical carcinoma and 2 with normal, respectively. Compared with the HPV18 reference sequence, the nucleotide variation rate of L1 was 98.28% (57/58). The L1 variants were divided into 12 different groups denoted as 18ME01–18ME12, which were also submitted to GenBank (accession numbers PP408305 − PP408316). In L1 sequences, 20 nucleotide substitutions were observed, including 11 non-synonymous variants G5503A (R25Q), A5520G (S31G), A5580G (R51G), C5769T (P114S), C5875A (T149N), C5920T (A164V), A6430C (Q334P), G6921A (E498K), A6970C (K514T), A7045C (K539T), C7062T (R545C) and 9 synonymous variants A5774C, A5832C, T5882C, A5924C, T5942G, A6125G, A6401G, A6404G, A6878C. The prevalent variant was G5503A (R25Q) (57/58). After further study, the non-synonymous variant G5503A (R25Q) was found in all variation groups of HPV18 L1. A5774C, T5882C, A6125G, A6404G, A6878C, G6921A and C7062T were novel variants. One non-synonymous variant A6970C (K514T) occurred encoding the alpha helix, three non-synonymous variants (S31G, P114S and A164V) were found encoding the strand. The results were shown in Table 3.

### Phylogenetic analysis of HPV18 E6-E7 and L1

**Fig. 1:**
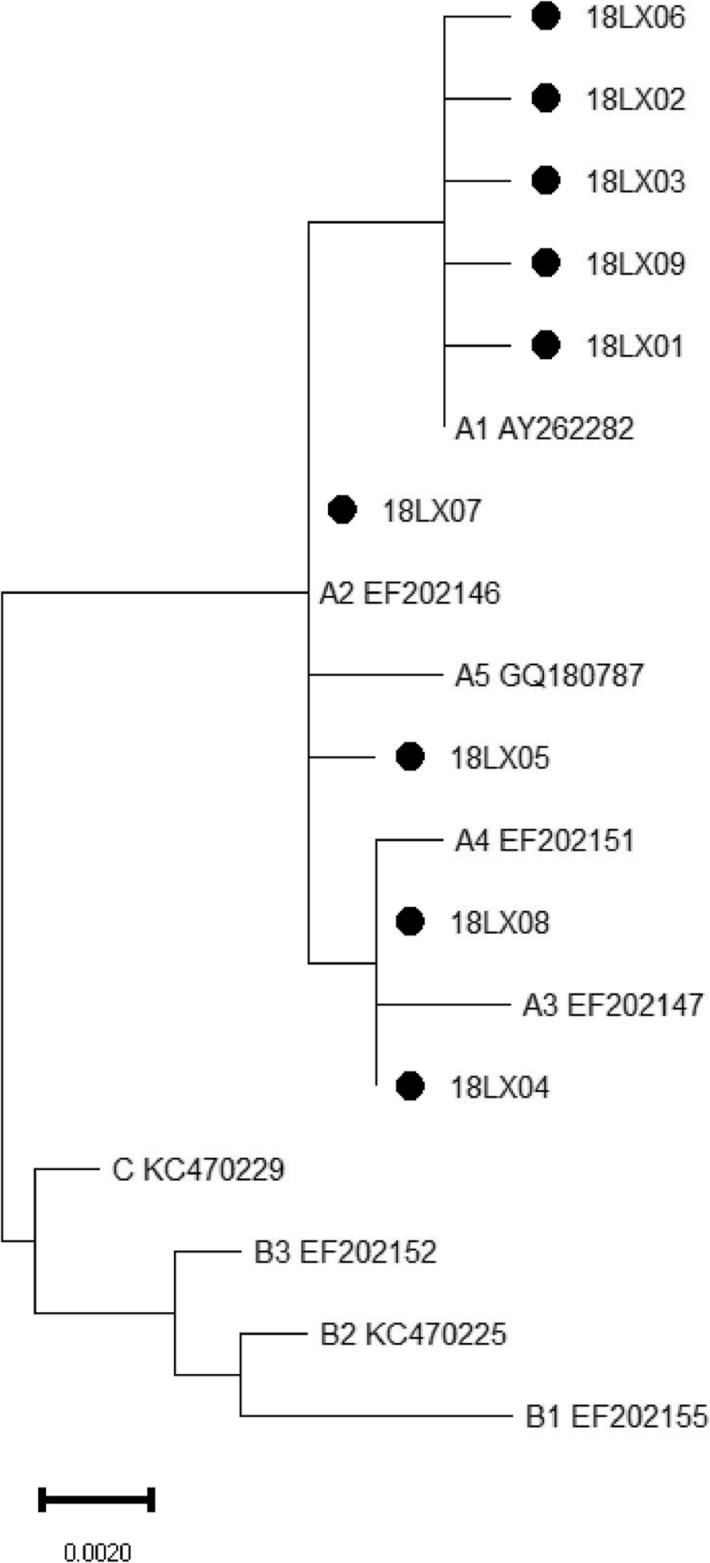
The phylogenetic tree of HPV18 E6-E7. A1–5, B1–3 and C represented the reference sequences of sublineages, and the others were variant sequences

**Fig. 2:**
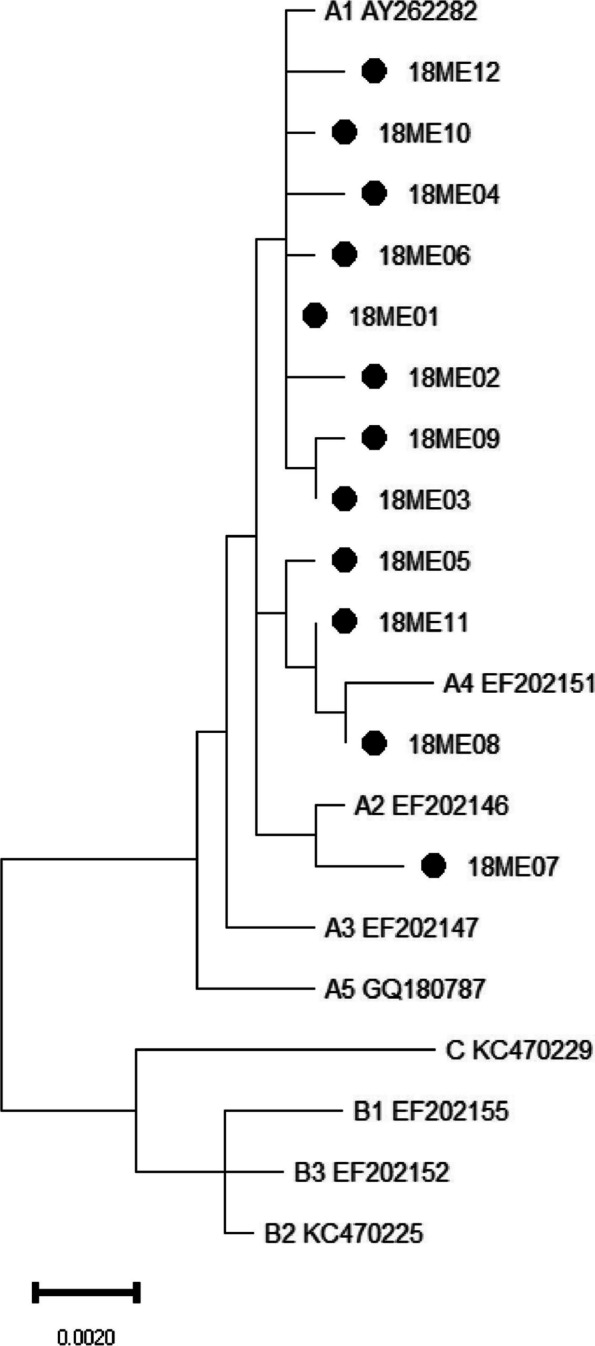
The phylogenetic tree of HPV18 L1. A1–5, B1–3 and C represented the reference sequences of sublineages, and the others were variant sequences

Phylogenetic analysis of HPV18 E6-E7 nucleotide sequences was performed, including 9 isolates and 9 reference sequences. To HPV18 L1, phylogenetic analysis was based on 12 isolates and 9 reference sequences. The phylogenetic trees of the E6-E7 and L1 sequences of HPV18 were shown in Figs. 1 and 2, respectively. 9 E6-E7 isolates (18LX01–18LX09) and 12 L1 isolates (18ME01–18ME12) were all classified into the A variant lineage.

### Selective pressure analysis

No positively selected sites were found in E6 and E7 sequences, and the results were shown in Table 4. 25R, 31S, 51R, 114P, 149 T, 164A, 334Q, 498E, 514 K, 539 K and 545R in L1 was initially shown to be the possible positive selection site through the analysis of the Codeml program in pamlX. However, after more rigid analysis using Bayes Empirical Bayes (BEB) analysis, no statistically significant positive selection site existed in L1, as shown in Table 4.

**Table 4:** Positively selected sites of HPV18 E6, E7, and L1

**Gene**	**Models**	**InL**	**Estimates of parameters**	**2Δl**	**Positively** **selected sites**
E6	M7	-673.299224	*p* = 4.23903, q = 99.00000		NA
	M8	-673.303679	p0 = 0.95569, *p* = 0.00654, q = 2.97246, p1 = 0.04431, ω = 1.00000	0.00891	NA
E7	M7	-437.438956	*p* = 52.05730, q = 99.00000		NA
	M8	-437.438731	p0 = 0.99999, *p* = 51.66086, q = 99.00000, p1 = 0.00001, ω = 1.00000	0.00045	NA
L1	M7	-2445.592068	*p* = 0.00500, q = 0.01184		NA
	M8	-2444.945253	p0 = 0.91343, *p* = 0.00500, q = 1.96135, p1 = 0.08657, ω = 4.03016	1.29363 *p* > 0.05	NA

The positively selected sites were identified with posterior probability ≥ 0.9 using Bayes empirical Bayes (BEB) approach

*Abbreviations*: *InL* log‐likelihood difference between the two models, *2Δl* twice the log‐likelihood difference between the two models, *NA* Not allowed, *NS* the sites not reaching the significant level

### B cell epitope prediction of HPV18 L1 protein

**Fig. 3:**
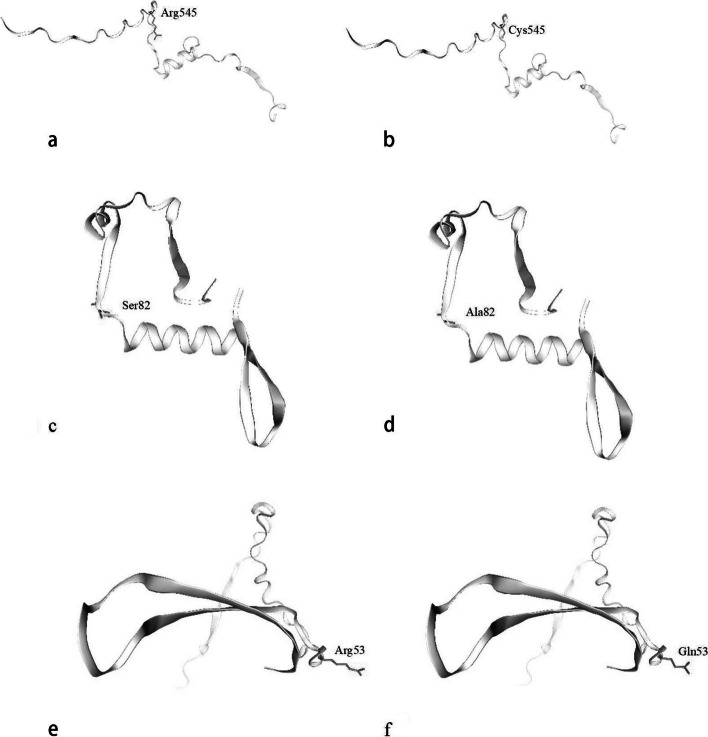
Conformational analysis of the epitopes. **a** Epitope conformation of Arg545; **b** Epitope conformation of Cys545; **c** Epitope conformation of Ser82; **d** Epitope conformation of Ala82; **e** Epitope conformation of Arg53; **f** Epitope conformation of Gln53

Based on the amino acid reference sequence and variant sequence of HPV18 L1, the prediction of epitope was done in order to find the potential B cell epitopes of HPV prophylactic vaccine. The prediction results were shown in Table 5. Only B cell epitopes with a score greater than 0.9 were listed. According to the rank, the most potent B cell epitopes of L1 was 539–554 KPTIGPRKRSAPSATT. In addition, amino acid variants may result in changes in prediction scores. For example, for the reference sequence of L1, the scores of 539–554 KPTIGPRKRSAPSATT was 0.93. But for amino acid variant R545C, the epitopes score of 539–554 KPTIGPCKRSAPSATT decreased to 0.92. Through the conformation analysis of HPV18 L1 epitopes containing the substitution of R545C, the results were shown in Fig. 3a and b. It is obvious that there was very minor conformational change at the place where the substitution took place.

**Table 5:** B cell epitope prediction of HPV18 L1 reference and variant sequences

**Reference sequence**				**Variants sequence**			
**Rank**	**Sequence**	**Start position**	**Score**	**Rank**	**Sequence**	**Start position**	**Score**
1	**KPTIGPRKRSAPSATT**	539	0.93	1	**KPTIGPCKRSAPSATT**	539	0.92
2	CQSICKYPDYLQMSAD	286	0.92	1	CQSICKYPDYLQMSAD	286	0.92
3	DNTVYLPPPSVARVVN	69	0.91	2	DNTVYLPPPSVARVVN	69	0.91
4	KFLVQAGLRRKPTIGP	529	0.90	3	KFLVQAGLRRKPTIGP	529	0.90
4	GTACKSRPLSQGDCPP	233	0.90	3	GTACKSRPLSQGDCPP	233	0.90
4	VFRVQLPDPNKFGLPD	133	0.90	3	VFRVQLPDPNKFGLPD	133	0.90
4	KVSAYQYRVFRVQLPD	125	0.90	3	KVSAYQYRVFRVQLPD	125	0.90

Bold parts indicate that the score of the sequences has changed

### T cell epitope prediction of HPV18 E6 and E7 proteins

Based on the amino acid reference sequence and variant sequence of HPV18 E6 and E7, the T cell epitope prediction was performed to find the potential T cell epitopes of HPV therapeutic vaccine. The prediction results were shown in Table 6 and Table 7. Only optimal epitopes or T cell epitopes containing variant amino acid sites were listed. 84 − 92 SVYGDTLEK for HLA‐A*11:01 (0.01) and 47 − 56 FAFKDLFVVY for HLA‐B*46:01 (0.01) showed the best binding affinity for E6. 7 − 15 TLQDIVLHL for HLA‐A*02:01 (0.01) indicated the best binding affinity for E7. The mutation of S82A of E6 resulted in new epitopes, such as 74 − 82 RIRELRHYA for HLA‐A*30:01, 81 − 90 YADSVYGDTL for HLA‐C*08:01, 72 − 86 YSRIRELRHYADSVY for DRB1*12:01 and 75 − 89 IRELRHYADSVYGDT for DRB1*15:01. The above four new epitopes all decreased the percentile and increased the binding affinity to T cells. The conformational analysis results of HPV18 E6 epitopes containing S82A substitution were shown in Fig. 3c and d. The mutation of R53Q of E7 led to new epitopes, such as 46 − 54 HQHLPARQA for HLA‐A*30:01 and 53 − 62 QAEPQRHTML for HLA‐C*01:02. And it made the percentile values change, up or down, resulting in the divergent binding affinities. For example, 52 − 61 RRAEPQRHTM for HLA‐B*13:01, the reference is 3.3, but the variant is 0.02, more compacted binding with T cell. The epitopes 53 − 62 RAEPQRHTML for HLA‐C*01:02 of E7 changed because of the mutation of R53Q, percentile value increasing by 0.09.The conformational analysis results of HPV18 E7 epitopes containing R53Q substitution were shown in Fig. 3e and f.

**Table 6:** T cell epitopes of HPV18 E6 reference and variant sequences

MHC allele	Start	End	E6 Reference sequence	PR	Start	End	E6 Variants sequence	PR
HLA‐A*02:01	24	33	SLQDIEITCV	0.09	24	33	SLQDIEITCV	0.09
HLA‐A*11:01	84	92	SVYGDTLEK	0.01	84	92	SVYGDTLEK	0.01
HLA‐A*24:02	85	93	VYGDTLEKL	0.02	85	93	VYGDTLEKL	0.02
HLA‐A*30:01	84	92	SVYGDTLEK	0.07	84	92	SVYGDTLEK	0.07
HLA‐A*30:01	74	82	**RIRELRHYS**	0.1	74	82	**RIRELRHYA**	0.02
HLA‐A*33:03	70	79	DFYSRIRELR	0.06	70	79	DFYSRIRELR	0.06
HLA‐B*13:01	37	45	TVLELTEVF	0.2	37	45	TVLELTEVF	0.2
HLA‐B*15:01	25	34	LQDIEITCVY	0.15	25	34	LQDIEITCVY	0.15
HLA‐B*40:01	4	14	FEDPTRRPYKL	0.15	4	14	FEDPTRRPYKL	0.15
HLA‐B*46:01	47	56	FAFKDLFVVY	0.01	47	56	FAFKDLFVVY	0.01
HLA‐B*58:01	36	45	KTVLELTEVF	0.15	36	45	KTVLELTEVF	0.15
HLA‐C*01:02	13	21	KLPDLCTEL	0.01	13	21	KLPDLCTEL	0.01
HLA‐C*03:04	47	55	FAFKDLFVV	0.22	47	55	FAFKDLFVV	0.22
HLA‐C*07:02	73	81	SRIRELRHY	0.06	73	81	SRIRELRHY	0.06
HLA‐C*08:01	81	90	**YSDSVYGDTL**	0.14	81	90	**YADSVYGDTL**	0.07
DPB1*05:01	122	136	NEKRRFHNIAGHYRG	0.29	122	136	NEKRRFHNIAGHYRG	0.29
DQB1*03:01	53	67	FVVYRDSIPHAACHK	2.1	53	67	FVVYRDSIPHAACHK	2.1
DRB1*09:01	53	67	FVVYRDSIPHAACHK	4.4	53	67	FVVYRDSIPHAACHK	4.4
DRB1*12:01	72	86	**YSRIRELRHYSDSVY**	4.4	72	86	**YSRIRELRHYADSVY**	3.40
DRB1*15:01	75	89	**IRELRHYSDSVYGDT**	0.88	75	89	**IRELRHYADSVYGDT**	0.84

Bold parts indicate that the PR of the sequences has changed

*A separator that separates the front genetic region from the back digital region

**Table 7:** T cell epitopes of HPV18 E7 reference and variant sequences

MHC allele	Start	End	E7 Reference sequence	PR	Start	End	E7 Variants sequence	PR
HLA‐A*02:01	7	15	TLQDIVLHL	0.01	7	15	TLQDIVLHL	0.01
HLA‐A*11:01	6	14	ATLQDIVLH	0.26	6	14	ATLQDIVLH	0.26
HLA‐A*24:02	88	96	QLFLNTLSF	0.46	88	96	QLFLNTLSF	0.46
HLA‐A*30:01	43	51	GVNHQHLPA	1.7	43	51	GVNHQHLPA	1.7
HLA‐A*30:01	46	54	**HQHLPARRA**	1.8	46	54	**HQHLPARQA**	1.2
HLA‐A*33:03	44	52	VNHQHLPAR	0.62	44	52	VNHQHLPAR	0.62
HLA‐B*13:01	7	15	TLQDIVLHL	0.02	7	15	TLQDIVLHL	0.02
HLA‐B*13:01	52	61	**RRAEPQRHTM**	3.3	52	61	**RQAEPQRHTM**	0.02
HLA‐B*15:01	88	96	QLFLNTLSF	0.05	88	96	QLFLNTLSF	0.05
HLA‐B*15:01	52	61	**RRAEPQRHTM**	4.3	52	61	**RQAEPQRHTM**	0.03
HLA‐B*40:01	54	62	AEPQRHTML	0.2	54	62	AEPQRHTML	0.2
HLA‐B*46:01	78	86	SSADDLRAF	0.06	78	86	SSADDLRAF	0.06
HLA‐B*58:01	78	86	SSADDLRAF	0.28	78	86	SSADDLRAF	0.28
HLA‐C*01:02	53	62	**RAEPQRHTML**	0.1	53	62	**QAEPQRHTML**	0.19
HLA‐C*03:04	53	61	**RAEPQRHTM**	0.03	53	61	**QAEPQRHTM**	0.05
HLA‐C*07:02	52	61	**RRAEPQRHTM**	0.16	52	61	**RQAEPQRHTM**	3.3
HLA‐C*08:01	53	61	**RAEPQRHTM**	0.05	53	61	**QAEPQRHTM**	0.09
DPB1*05:01	81	95	DDLRAFQQLFLNTLS	2.2	81	95	DDLRAFQQLFLNTLS	2.2
DQB1*03:01	74	88	LVVESSADDLRAFQQ	6.6	74	88	LVVESSADDLRAFQQ	6.6
DRB1*09:01	10	24	DIVLHLEPQNEIPVD	6.9	10	24	DIVLHLEPQNEIPVD	6.9
DRB1*12:01	10	24	DIVLHLEPQNEIPVD	3.7	10	24	DIVLHLEPQNEIPVD	3.7
DRB1*15:01	79	93	SADDLRAFQQLFLNT	2.4	79	93	SADDLRAFQQLFLNT	2.4

Bold parts indicate that the PR of the sequences has changed

*A separator that separates the front genetic region from the back digital region

## Discussion

Cervical cancer is the fourth most common cancer in women globally and an important public health problem [24]. According to the latest data from the World Health Organization, there were around 660 000 new cases and around 350 000 deaths in 2022. It is generally believed that persistent HR-HPV infection is the most important cause of cervical cancer [25]. Among all HPV types, the carcinogenicity of HPV18 is second only to HPV16, accounting for approximately 12% of cervical squamous cell carcinomas and 37% of adenocarcinomas worldwide [26]. In our previous study, the infection rate of HPV18 was 5.92% in Jingzhou area [27]. In this study, the sequences of HPV18 E6, E7 and L1 isolated from women in central China were analyzed.

It has been speculated that the differences in nucleotide variants occurring in HPV intra-type variants result in both function and pathogenicity divergence [28, 29]. E6 and E7 oncoproteins show strong correlation with cervical cancer and participate in the immortalization of cervical cancer cells [30]. In this study, E6-E7 was more conservative than L1. The variation rates of E6-E7 and L1 were 16.13% (10/62) and 98.28% (57/58), respectively. HPV18 E6-E7 genes were very stable, and 52 (52/62, 83.87%) isolates were identical to the reference sequence. A482C, T485C, C549A and C751T variants of HPV18 E6-E7 have been reported in Northeast China [22]. A482C of E6 has been found in southwest China [31]. T485C and C549A variants of E6 have been reported in Henan Province, China [32]. In Korea, A482C, T485C, C549A and C751T variants ofHPV18 E6-E7 have been reported [21]. T485C, C549A and C751T variants have been reported in Indonesia, Suriname and the Netherlands [33]. The A482C, T485C, C549A, G747A, and C751T variants of HPV18 E6-E7 have been previously reported in Japanese women [34]. In this study, 9 variants were identified in HPV18 E6-E7 gene, of which 5 variants (A482C, T485C, C549A, G747A, C751T) were also found in other regions and 4 variants (T348G, T392C, T467G, C835T) were newly discovered. Due to the limited sequence number of 18LX01-18LX09, the association between specific nucleotide variants and cervical lesions was not suitable to analyze, which is compatible with the results before in China [31, 32].

The host's cellular immune response may be affected by mutations in the L1 gene, which may have an impact on vaccine effectiveness [35]. In this study, we observed 20 variation sites in HPV18 L1, including 13 previously reported variants and 7 novel variants. In all isolates containing variants, G5503A was present in HPV18 L1, consistent with the report in Henan province, China [32]. HPV18 L1 variants identified in this study have also been found in other regions. In Northeast China, G5503A, C5875A, C5920T, A6401G, A6430C and A7045C variants have been reported [22]. In Taizhou area, China, the variants identified in HPV18 L1 are G5503A, A5580G, A5832C, C5875A, C5920T, A5924C, T5942G, A6401G, A6430C and A7045C [23]. In Southwest China, G5503A variant is found in HPV18 L1 [36]. In the Netherlands, A5520G, C5769T and A6970C variants are in HPV18 L1 [37].

Nucleotide sequence differences were 1–10% in the lineages of HPV type, and 0.5–1% in the sublineages [28]. HPV18 variants are originally grouped into European (E), Asian-Amerindian (AA) or African (AFR) lineages according to E6-E7, L1 or LCR sequences [26]. The whole viral genome sequencing approach classifies HPV18 into three major lineages (A, B, and C) and additional sublineages (A1 to A5 and B1 to B3), and can be largely translated into the historical nomenclature (A1 and A2 = AA, A3 to A5 = E, and B and C = AFR) [16]. In this study, all the HPV18 E6-E7 and L1 variant sequences only distributed in A lineage. It is consistent with the report that the most common HPV18 variant lineage in Northeast China was AA (81.5%), followed by E (18.5%) [22].

Most of the positive selection mutations are common non-synonymous mutations and widely adapt to the environment [38]. The selective pressure at protein level is measured by the non-synonymous substitution rate ratio (ω). An ω greater than 1 indicates that non-synonymous mutations are more adaptable and have a higher fixed rate in the population than synonymous mutations [39]. In this study, there was no evidence of positive selection in the sequence alignment of HPV18 E6, E7 and L1 genes. This was consistent with no positive selection of HPV18 E6/E7 gene reported in southwest China [31].

Both T cells and B cells are involved in adaptive immunity, and the memory and effector functions of T cells and B cells depend on epitope recognition of pathogens [40]. As the smallest structural and functional unit of an antigen, epitope can bind specifically to antibodies or antigen receptors and is a key site for adaptive immune activation [41]. The researches on diversity of epitope play a pivotal role in disease diagnosis, vaccine design, and immunotherapy. Currently, all available prophylactic HPV vaccines are based on VLPs that self-assemble spontaneously from the L1 major capsid protein [42]. In this study, we predicted the B cell epitopes of HPV18 using the reference sequence protein of L1 and the L1 variant sequence in central China, and obtained 7 optimal epitopes. In the variant sequence, the epitope prediction score changes due to amino acid changes. For example, R545C of L1 resulted in a decrease of 0.1 in the B cell epitope score. Therefore, the variation should be considered in the study of epitope. The reason behind the score change could be that the variation alters hydrophilicity, whereas the hydrophilic region of the protein is associated with antigenicity [43].

The HPV prophylactic vaccine does not provide effective treatment for an already infected organism. However, therapeutic HPV vaccines are designed to induce a virus-specific T cell response in vivo to combat pre-existing HPV infections and lesions [15]. E6 and E7 oncoproteins are important proteins in the development of HPV-related malignancies, and are the two most important target antigens for the development of HPV therapeutic vaccines [44]. In this study, T cell epitopes of HPV18 E6 and E7 proteins were predicted, and the optimal epitope sequences were obtained. The predicted score of some epitope sequences changed due to amino acid changes, such as S82A in 74 − 82 RIRELRHYS in E6, which caused PR to decrease from 0.1 to 0.02, and R53Q in 52 − 61 RRAEPQRHTM in E7, which caused PR to increase from 0.16 to 3.3. In immunogenicity, E6/E7 amino acid substitution affects the immune response by presenting different peptides to specific T cells through polymorphic HLA molecules [43].

In this study, through the prediction analysis of several common MHC alleles in the Chinese Han population, it was found that some T cell epitopes showed excellent affinity for different alleles, and no mutations occurred in these epitopes. For example, the epitope 84 − 92 SVYGDTLEK of E6 was the optimal epitope in alleles HLA‐A*11:01 and HLA‐A*30:01. E7 epitope 7 − 15 TLQDIVLHL was the optimal epitope in alleles HLA-A *02:01 and HLA-B *13:01. The amino acid changes of HPV E6 and E7 may affect the epitopes and the relevant the immune recognition of HPV-infected cells [45]. However, the optimal epitope for therapeutic vaccines is selected from the same region of the reference sequence and variant sequence [46]. The information of these epitopes can provide reference for vaccine research. However, due to the limitation of the epitope prediction accuracy, more in vitro and in vivo studies are needed to further verify.

## Conclusion

In this study, the genetic variation of HPV18 E6, E7 and L1 genes was studied in Jingzhou area. There were 29 variants in the HPV18 E6, E7 and L1 gene sequences, including 11 new variants. Some of these variants resulted in corresponding amino acid sequence changes and had an effect on the predicted T or B cell epitopes encoded by the virus. All HPV18 variants belonged to A lineage. This study provided useful data for the epidemiological characteristics, immunotherapy, and the development of novel prophylactic and therapeutic vaccines of HPV18 in central China.

## Data Availability

The data generated during the current study are available in the NCBI repository (Home—Nucleotide—NCBI (nih.gov)). The sequence data were submitted to GenBank with accession numbers OR880987–OR880995 and PP408305 − PP408316.

## Abbreviations

HPV18: Human papillomavirus type 18
HR-HPV: High-risk human papillomavirus
URR: Upstream regulatory region
VLPs: Virus-like particles
PCR: Polymerase chain reaction
MHC: Major histocompatibility complex
HLA: Human leukocyte antigen
The amino acid acronyms R, A, G, F, C, D, N, E, I, L, V, Q, P, S, K and T: Arginine, alanine, glycine, phenylalanine, cysteine, aspartic acid, asparagine, glutamic acid, isoleucine, leucine, valine, glutamine, proline, serine, lysine and threonine
